# Exacerbation of Very Late-Onset Darier Disease With COVID-19 Infection: A Case Report

**DOI:** 10.7759/cureus.43353

**Published:** 2023-08-11

**Authors:** Samar Sajid, Rahul Adwani, Malik Ahsan Safdar, Umer Talal, Muhammad Imtanan Fazal

**Affiliations:** 1 Medicine, Dow University of Health Sciences, Karachi, PAK; 2 Medicine, Services Hospital Lahore, Lahore, PAK

**Keywords:** rash, keratosis follicularis, atp2a2, covid 19, darier’s disease

## Abstract

Darier disease is an uncommon hereditary skin disorder characterized by the presence of hyperkeratotic papules and plaques affecting seborrheic areas. The uniqueness of this case lies in the exceptionally late-onset pattern of Darier disease, involving an 82-year-old female patient, and its correlation with COVID-19 infection. The patient had a history of a scaly and itchy rash limited to her arms, initially misdiagnosed as dermatitis, which persisted and worsened over three months. The manifestation of classical features of Darier disease coincided with her recent contraction of COVID-19. This instance emphasizes the varying manifestations of Darier disease that appear very late in life, which could result from new mutations or partial penetrance. Additionally, this case points out the potential worsening of Darier disease when combined with a COVID-19 infection. It highlights the need to be aware of atypical clinical progressions and the potential for increased severity of skin disorders during COVID-19. More studies are essential to grasp the relationship between COVID-19 and inherited skin conditions, aiming to improve patient treatment and care approaches.

## Introduction

Darier disease was first described by Prince Marrow in 1886 and later independently by Darier and White in 1889, with Darier naming it “psorospermose folliculaire vegetante” [1]. Darier disease, also known as keratosis follicularis, is a dermatological disorder characterized by heterozygous mutations in the ATP2A2 gene, resulting in complete penetrance and variable expressivity. These mutations can be inherited in an autosomal dominant manner or occur sporadically. Interestingly, two-thirds of reported cases are of the sporadic form, affecting individuals with no family history of the disorder. The ATP2A2 gene is responsible for producing the enzyme SERCA2, which plays a crucial role in regulating calcium ions within the endoplasmic reticulum and sarcoplasmic reticulum. Mutations in this gene lead to a deficiency of the functional SERCA2 enzyme, causing a disruption in calcium levels within the endoplasmic reticulum and subsequent dysfunction. As a result, the role of calcium signaling in cell-to-cell adhesion and desmosome adhesive strength is affected. Keratinocytes, which are held together by calcium-dependent desmosomes, experience abnormalities in adhesion (acantholysis) and differentiation (dyskeratosis) due to the altered SERCA2 function [2].

Clinically, the disease is characterized by the persistent eruption of hyperkeratotic papules and plaques, primarily affecting seborrheic areas, along with nail abnormalities and mucosal involvement [3]. Most patients typically develop initial lesions during their second decade of life, and the estimated prevalence is one to four per 100,000 people [4]. While both men and women are affected, there has been a description of a male predominance in some cases [5]. This case report features an 82-year-old female patient with very late-onset Darier disease and its potential exacerbation with COVID-19 infection.

## Case presentation

**Figure 1:**
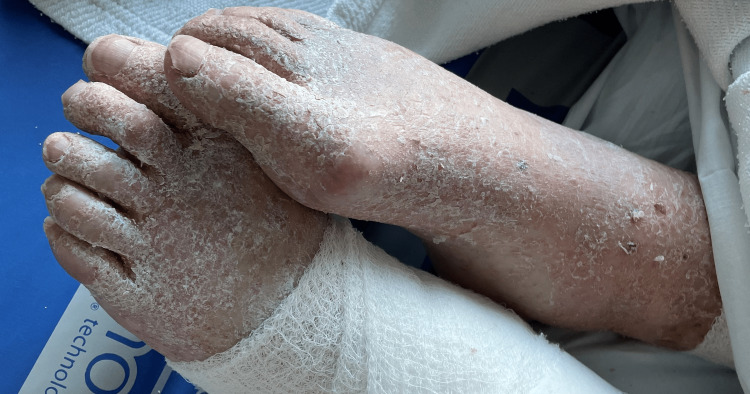
Silver scaly rash with a sandpaper-like texture on the dorsum of feet, nail beds showing unequal texture, longitudinal stripes, and nicking of margins.

**Figure 2:**
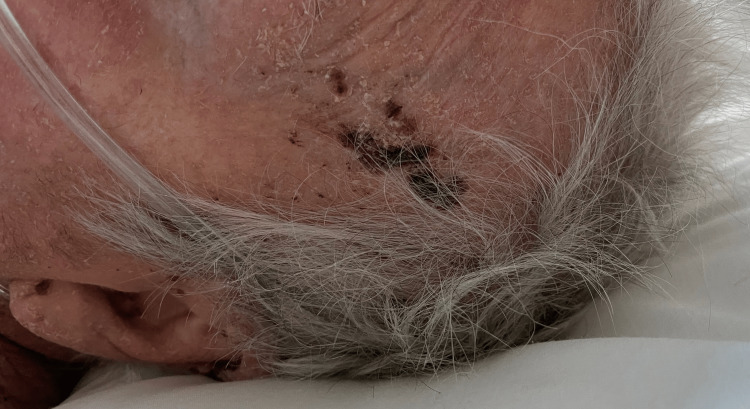
Brown-colored papules on the scalp, a few mm in size, clearly localized by healthy skin.

**Figure 3:**
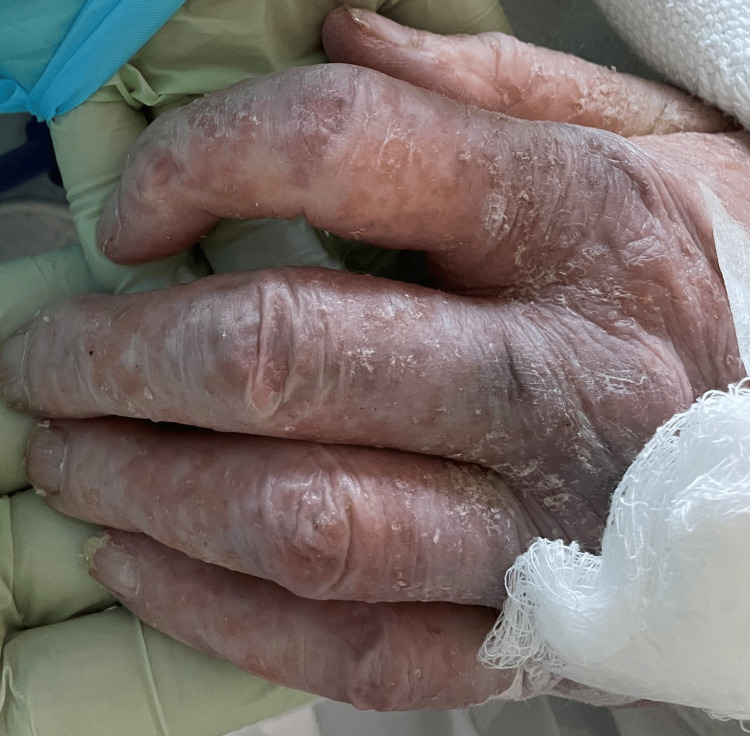
Xerotic excoriating rash on the dorsum of hands with areas of eczema, nail bed reveals unequal textures and white streaks with nicking of margins.

An 82-year-old Caucasian woman presented to our facility with respiratory distress, altered consciousness, and a worsened rash that had developed over the past two days. She tested positive for COVID-19. Physical examination revealed the patient was incoherent, with a Glasgow Coma Scale (GCS) score of 8/15. Auscultation of her chest indicated the presence of rhonchi and crepitations in the middle lobe of her right lung. The skin examination revealed a diffuse erythematous body rash with areas of scaliness, scab formation, and a sandpaper-like texture, particularly prominent on the face, abdomen, and dorsum of the feet (Figure 1). Additionally, severe involvement of the lower abdomen, lower back, and intertriginous areas (axilla, inframammary folds, and groin) presented as a purpuric weeping rash. The lower back rash exhibited purulent discharge and bleeding. The lesions emitted a strong odor. Brown-colored papules were observed on the face and scalp, clearly localized by healthy skin (Figure 2). The nails of her hands and feet showed unequal textures, white streaks on nail beds, and V-shaped nicks on the free margins (Figure 3). The oral mucosa was also affected, displaying white papules.

The patient reported that the lesions had begun three months prior as a mild itchy rash on her arms, initially managed as dermatitis but was unresponsive to steroids and gradually worsened in intensity and distribution. She experienced pruritus and an exacerbation of symptoms with exposure to sunlight, heat, friction, and moisture. No family members had been diagnosed with hereditary diseases or had similar lesions. There was no known exposure to new drugs, over-the-counter medications, different clothing, cosmetics, or skin care products. Moreover, she had not recently traveled or been in contact with a sick person. The patient's medical history included a 28-year history of smoking one pack of cigarettes per day, with smoking cessation at the age of 50. Her comorbidities comprised acute kidney injury, depression, hypertension, hyperlipidemia, gastroesophageal reflux disease, and hypothyroidism.

Hematologic tests showed leukopenia, elevated levels of amino transaminase, lactate dehydrogenase, ferritin, and C-reactive protein, and low albumin. Renal function tests indicated deranged results and thyroid-stimulating hormone (TSH) was within the normal range. Cultures from blood and skin samples showed no growth, and the Tzanck smear revealed no evidence of herpesvirus infection. The diagnosis of Darier disease was established by the referring dermatologist based on skin biopsy findings of acanthosis, hyperkeratosis, and papillomatosis. COVID-19 was confirmed through polymerase chain reaction (PCR) testing.

She was managed in the intensive care unit with fluids, non-invasive ventilation (pressure support of 5 cm H_2_O and a PEEP of 7 cm H_2_O), daptomycin (350 mg once a day), azithromycin (500 mg once a day), ceftriaxone (2 g once a day), dexamethasone (6 mg once a day), remdesivir (200 mg on day one followed by 100 mg from day 2 to day 5), enoxaparin (60 mg once a day), acetretin (20 mg once a day), and topical triamcinolone. However, the patient expired after three days due to acute respiratory distress syndrome.

## Discussion

This case sheds light on the variable expressivity of Darier disease, particularly its very late clinical onset, which challenges conventional assumptions about its hereditary nature. The absence of a positive family history, in this case, points to the possibility of a new mutation or incomplete penetrance, where milder forms of the disease might have gone unnoticed among family members.

The exact cause of late-onset Darier disease remains unclear, and there have been no definitive studies pinpointing the underlying mechanisms. It is suggested that the disease's diverse symptoms, such as vesicobullous, cornifying, comedonal, acral, hemorrhagic, and linear types, could be attributed to genetic background and environmental influences, leading to varying severity among individuals within the same family [3]. Factors, like sunlight exposure, mechanical trauma, heat, sweat, humidity, lithium, and oral steroids, have been linked to symptom exacerbation [4].

Due to its rarity, misdiagnosis of Darier disease as other skin conditions, such as eczema, seborrheic dermatitis, and Hailey-Hailey disease, is not uncommon. Acrokeratosis verruciformis of Hopf, sharing the same mutation as Darier disease, presents with similar papules on the extremities but lacks involvement in sebaceous areas, flexural sites, and oral mucosa [6]. Notably, a theory suggests a potential association between Darier disease and neuropsychiatric disorders due to the high expression of calcium pumps in brain tissue. The condition has been linked to psychiatric conditions like schizophrenia, intellectual impairment, bipolar disorder, and depression, independent of family history and disease severity, indicating a connection to the ATP2A2 mutation itself [7].

Treatment options for Darier disease focus on managing symptoms, as there is no specific cure. Topical corticosteroid creams, retinoids, emollients, and sunscreen are commonly used. In more extensive forms, oral acitretin may be prescribed, although it comes with significant adverse effects including arthralgias, myalgias, psychiatric disorders, etc. [8]. Protection from trauma and sunlight is crucial, and oral retinoids are used to reduce hyperkeratosis and improve papule appearance and odor. In some cases, oral antibiotics and antiviral medications are necessary to address secondary infections. Various therapeutic options, including dermabrasion, electrosurgery, laser ablation, photodynamic therapy, and surgical excision, have been listed in the literature [5].

## Conclusions

This case highlights the rare occurrence of very late-onset Darier disease, presenting in an 82-year-old female patient and coinciding with a COVID-19 infection. The absence of a positive family history raises the possibility of new mutations or incomplete penetrance contributing to the disease's variability. The complex interplay of genetic and environmental factors may account for the diverse symptomatology observed in Darier disease. Misdiagnoses are common due to their rarity and resemblance to other skin conditions. Additionally, the association with neuropsychiatric disorders adds to the complexity of management. Further research is needed to elucidate the underlying mechanisms of late-onset Darier disease and its potential exacerbation in the context of COVID-19. Enhancing our understanding will improve patient care and treatment strategies for this uncommon dermatological disorder.

## References

[1] Engin B, Kutlubay Z, Erkan E, Tüzün Y (2015). Darier disease: a fold (intertriginous) dermatosis. Clin Dermatol.

[2] Sakuntabhai A, Ruiz-Perez V, Carter S (1999). Mutations in ATP2A2, encoding a Ca2+ pump, cause Darier disease. Nat Genet.

[3] Burge SM, Wilkinson JD (1992). Darier-White disease: a review of the clinical features in 163 patients. J Am Acad Dermatol.

[4] Cooper SM, Burge SM (2003). Darier's disease: epidemiology, pathophysiology, and management. Am J Clin Dermatol.

[5] Suryawanshi H, Dhobley A, Sharma A, Kumar P (2017). Darier disease: a rare genodermatosis. J Oral Maxillofac Pathol.

[6] Dhitavat J, Macfarlane S, Dode L (2003). Acrokeratosis verruciformis of Hopf is caused by mutation in ATP2A2: evidence that it is allelic to Darier's disease. J Invest Dermatol.

[7] Dodiuk-Gad RP, Cohen-Barak E, Khayat M (2016). Darier disease in Israel: combined evaluation of genetic and neuropsychiatric aspects. Br J Dermatol.

[8] Ortiz NE, Nijhawan RI, Weinberg JM (2013). Acitretin. Dermatol Ther.

